# A genetic programming approach to oral cancer prognosis

**DOI:** 10.7717/peerj.2482

**Published:** 2016-09-21

**Authors:** Mei Sze Tan, Jing Wei Tan, Siow-Wee Chang, Hwa Jen Yap, Sameem Abdul Kareem, Rosnah Binti Zain

**Affiliations:** 1 Bioinformatics Program, Institute of Biological Sciences, Faculty of Science, University of Malaya, Kuala Lumpur, Malaysia; 2 Department of Mechanical Engineering, Faculty of Engineering, University of Malaya, Kuala Lumpur, Malaysia; 3 Department of Artificial Intelligence, Faculty of Computer Science & Information Technology, University of Malaya, Kuala Lumpur, Malaysia; 4 Oral Cancer Research & Coordinating Centre (OCRCC), Faculty of Dentistry, University of Malaya, Kuala Lumpur, Malaysia

**Keywords:** Genetic Programming, Oral cancer prognosis, Machine learning, Feature selection

## Abstract

**Background:**

The potential of genetic programming (GP) on various fields has been attained in recent years. In bio-medical field, many researches in GP are focused on the recognition of cancerous cells and also on gene expression profiling data. In this research, the aim is to study the performance of GP on the survival prediction of a small sample size of oral cancer prognosis dataset, which is the first study in the field of oral cancer prognosis.

**Method:**

GP is applied on an oral cancer dataset that contains 31 cases collected from the Malaysia Oral Cancer Database and Tissue Bank System (MOCDTBS). The feature subsets that is automatically selected through GP were noted and the influences of this subset on the results of GP were recorded. In addition, a comparison between the GP performance and that of the Support Vector Machine (SVM) and logistic regression (LR) are also done in order to verify the predictive capabilities of the GP.

**Result:**

The result shows that GP performed the best (average accuracy of 83.87% and average AUROC of 0.8341) when the features selected are smoking, drinking, chewing, histological differentiation of SCC, and oncogene p63. In addition, based on the comparison results, we found that the GP outperformed the SVM and LR in oral cancer prognosis.

**Discussion:**

Some of the features in the dataset are found to be statistically co-related. This is because the accuracy of the GP prediction drops when one of the feature in the best feature subset is excluded. Thus, GP provides an automatic feature selection function, which chooses features that are highly correlated to the prognosis of oral cancer. This makes GP an ideal prediction model for cancer clinical and genomic data that can be used to aid physicians in their decision making stage of diagnosis or prognosis.

## Introduction

Oral cancer, commonly known as mouth cancer, is the abnormal growth of cells found in the different regions of the mouth including the tongue, floor of the mouth, buccal mucosa (cheeks), and lips (National Institute of Dental and Craniofacial Research, 2016). According to the Oral Cancer Research and Coordinating Centre (OCRCC) in the University of Malaya, there are 350,000 new cases of oral cancer reported worldwide every year, bringing oral cancer to the rank of the 6th most common cancer in the world. In Malaysia, oral cancer is ranked the third most common cancer amongst the Indian Ethnic group and constitutes 13.2% of the cancer in Malaysia as reported by the National Cancer Registry (NCR) 2007 (Omar & Ibrahim Tamin, 2011).

The risk factor of oral cancer includes heavy use of tobacco, alcohol and betel quid. Besides that, the practice of imbalance diet i.e., the low intake of antioxidant-rich food may also cause the development of oral cancer. Some genetic factors, such as p53 and p63, have been found to be associated with oral cancer.

Various machine learning models, such as the Genetic Algorithm (GA), the Artificial Neural Network (ANN) and the Support Vector Machine (SVM), can actually “be trained” and “learn” from the given data in order to execute many functions. In the bio-medical field, most of these machine learning approaches are commonly used for pattern recognition, diagnosis and prognosis of diseases.

In this research, a machine learning approach, namely Genetic Programming (GP), is applied for oral cancer prognosis. Many recent studies have explored the functionality of GP in cells recognition (Dong, 2008; Nandi et al., 2006) or in gene expression profiling data (Hong & Cho, 2006; Mitra et al., 2006; Ho et al., 2006). However, the potential of GP in the classification of cancerous data for cancer prognosis purposes remain unclear. Based on our review, this is the first study that applied the GP technique in the oral cancer prognosis research. The main objective of this research is to study the feasibility of using GP as a feature selection and classification tool in a small dataset that contains limited number of data and variables. For benchmarking purposes, the prediction of 3-year oral cancer prognosis using GP is compared with SVM, a machine learning technique, and also logistic regression, a statistical method.

Most biomedical data samples are usually small in size but consist of many variables. This may cause over-fitting problem in the classification as the accuracy may be affected by the irrelevant features. Thus, feature selection steps should be taken before the classification steps in order to increase the accuracy of the results. Although the dataset involved in this research is small in size and does not contain many variables, the aim is to study the performance of GP the small subset of features in order to obtain the optimal result.

## Background Study

GP was first proposed by Koza in 1992 with the aim of evolving a population of programs instead of bit strings (Koza, 1992). GP continues the way of GA in dealing with the problem but increased the complexity of the adaptation structure in more general, hierarchical and dynamic way. According to Koza, GP reformulated the process of solving the problems of other machine learning methods by searching a highly fit individual program in a population of candidate programs. This space of searching consists of many functions and terminals, relevant to the problem domain. GP functions by searching the fittest individuals in the program.

GP breed populations of hundreds or thousands of computer programs using the Darwinian principal of survival and reproduction of the fittest, together with genetic operations during the process of evolution, namely, mutation and crossover. Thus, in general, GP solves the problems given by the combination of natural selection and genetic operations. There are many tasks that can be performed by using GP. In Mitra et al. (2006), the research was carried out to identify the nodal status in bladder using gene expression profiles analysis. This research used GP as a method to generate classifier rules in order to determine the nodal status. The authors identified genes *ICAM 1, MAP2K6, KDR, CDK8* and *ANXA5* involved in the expression of the positive node case.

Yu et al. (2007) proposed a method to applied GP in cancer expression profiling data to select gene features and build a molecular classifier by applying mathematical integration of the genes. The result of this research revealed that there is a set of highly significant feature genes that are repetitively associated with prostate cancer. Also, in this paper, the accuracy of GP classification is compared with other machine learning techniques, such as, compound covariate 3-Nearest Neighbor, Support Vector Machine, DLC and etc. The results showed that GP has a lower error rate (1.5% using 5 genes) as compared to other methods.

There are a some studies that use GP in Breast Cancer studies. In Vanneschi et al. (2011), the author compared the accuracy of several machine learning classifiers with GP using NKI Breast Cancer Dataset showing that GP performs significantly better than other methods in classifying breast cancer dataset and also comparable with the scoring-based method of the 70-gene signature. On the other hand, Guo & Nandi (2006) used GP as the feature selection method in their research. The authors compared the result of breast cancer diagnosis using the features generated by GP based on the criteria of various pattern recognition methods. The results of this research indicated that Modified Fisher Criterion-based GP (MP_GP) performed better than the other GP-based features extractor (Guo & Nandi, 2006).

Furthermore, GP is proved to be applicable in drug discovery. According to Archetti et al. (2007), GP is useful in predicting the pharmacokinetic of drug, which is the movement of drug in the human body. The authors reviewed the functionality of GP in the pharmacokinetics of drug, as well as comparing the ability of GP to predict oral bioavailability (RMSE of 30.1276), median oral lethal dose (RMSE of 1776.7700) and plasma-protein binding levels (RMSE of 34.6617) with other types of machine learning method.

Almost all of the studies involved GP in genomic and cancer data are done using microarray datasets which consists of microarray gene expression data and are larger in size. The only exception of GP application using non-microarray dataset was from Muni, Pal & Das (2006) which used several types of public datasets. However, only two datasets used are relevant to cancerous data which are Wisconsin Breast Cancer (WBC) and Wisconsin Diagnostic Breast Cancer (WDBC) (Blake & Merz, 1998). Both of these datasets included larger size of data (699 and 599 samples respectively) but with limited number of features (9 and 30 features respectively). The authors proposed a GP methodology which selected a better subset of features and used the features selected in constructing the classifier simultaneously. In the results of this study, the accuracy of the performance obtained using selected features dropped slightly (WBC-96.84% and WDBC-96.31%) while compared with the accuracy of using all features (WBC-97.42% and WDBC-97.26%). The authors claimed that the performance of their proposed method in using a smaller subset of features is comparable with that of all features and producing similar results.

In this research, however, a small locally collected dataset is used in order to review the performance of GP in small-sized dataset. We would like to test the feasibility of applied GP in a small dataset, whether GP could maintain its stability and obtain the good results that archived by using microarray datasets in the previous studies, and to test the suitability of GP as feature selection and classification tool in a small dataset.

## Materials and Method

### Data

A total of 31 oral cancer cases of 3-year prognosis were collected from the Malaysia Oral Cancer Database and Tissue Bank System (MOCDTBS) coordinated by the OCRCC, Faculty of Dentistry, University of Malaya. The procedures of data acquisition are the same as described in Chang et al. (2013). There are 17 features in the dataset, which are listed in the Table 1. Each case was followed up for three years from the date of the time when oral cancer was diagnosed. The outcome for each case is either dead or alive at the end of the three years.

**Table 1:** Features available in the oral cancer prognosis dataset.

**Feature**	
Age	*(Age)*
Ethnicity	*(Eth)*
Gender	*(Gen)*
Smoke	*(Smo)*
Drink	*(Dri)*
Chew	*(Chew)*
Site	*(Site)*
Histological differentiation of SCC	*(Diff)*
Pattern of invasion	*(Inv)*
Nodes	*(Nodes)*
PT	*(PT)*
PN	*(PN)*
Stage	*(Sta)*
Size	*(Size)*
Treatment	*(Tre)*
p53	(*p*53)
p63	(*p*63)

### Genetic Programming

**Figure 1:**
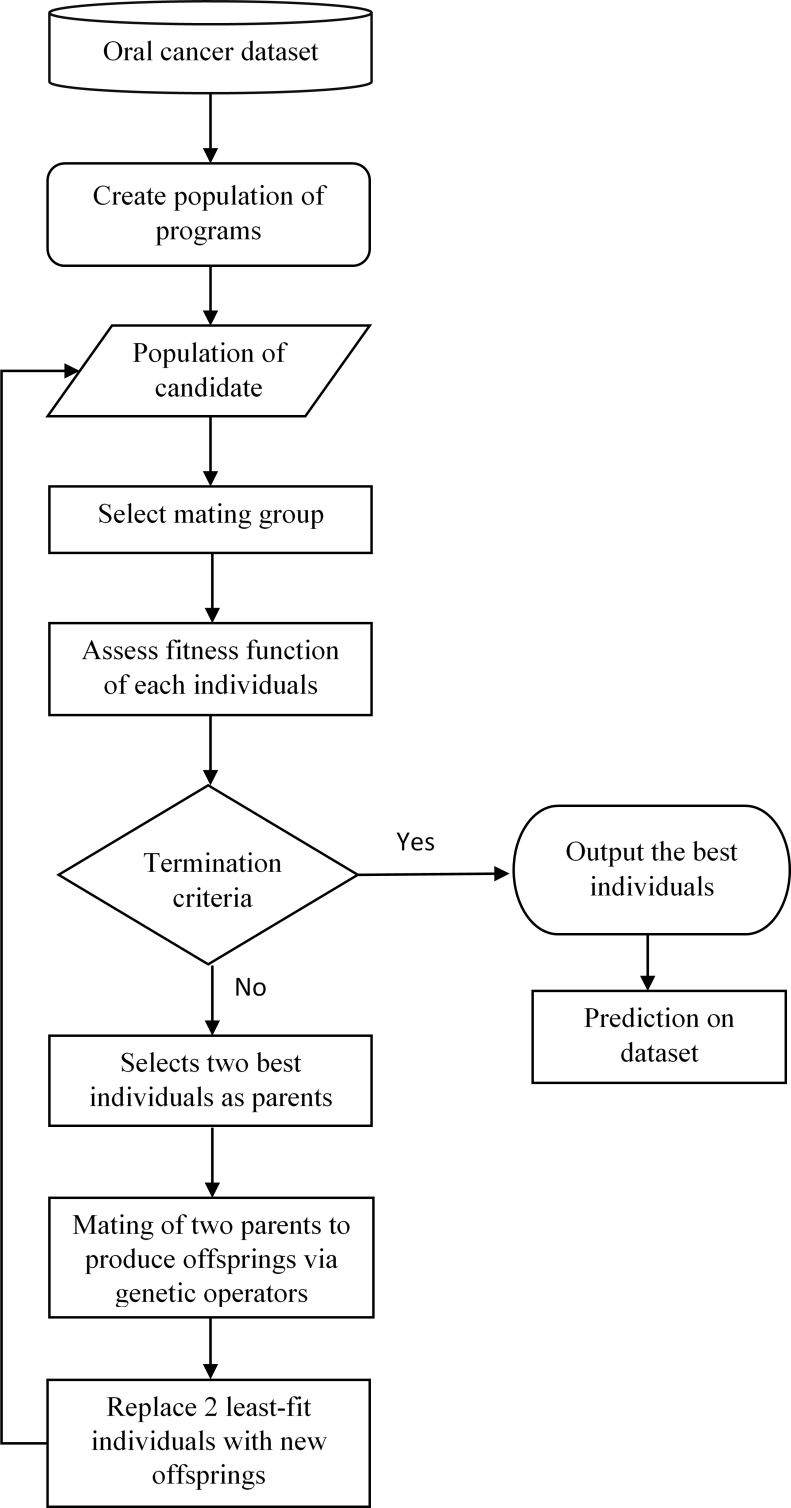
Framework of oral cancer prognosis with genetic programming.

Genetic Programming (GP) is a machine learning method that classifies a given dataset by simulating natural biological process, i.e., natural selection, crossover, and population dynamics in order to obtain the best relationships between each element in the system. GP calculates the fitness of each member in the population and generates generations of offspring to compete with the parents in order to obtain the best individuals in the populations (in this study, the best prediction result). Figure 1 shows the framework of the proposed oral cancer prognosis by using genetic programming.

Basically, GP is initiated by the interactions between the randomly selected inputs with the function operators, for instances, boolean and arithmetic operator, to compose an individual tree structure. The accumulations of these individual will form the initial population and the selection from these populations will form a small subgroup known as the “mating group.” The fitness function for each individual in this small population is evaluated. The two fittest individuals in this generation are selected for “mating” or act as the parents for the next generation to produce “offspring,” using *mutation* and *crossover* operators as the selective genetic operator. Then, a new generation is formed by replacing the generated offspring with the least-fit parents in the population until all the parent members in the population are fully replaced. This process of evaluating every member of the population, mating, and producing replacement offspring is looped over generations until a termination criterion, is reached, (i.e., the maximum number of generations) which is predefined firstly by the user of the program.

The aim of this genetic program is to obtain the best individual from the population with the best fitness value and the best result, which is the predicted outcome that generated by GP using the small oral cancer dataset that described earlier in this study. The predicted results were then compared with the three-year survival rate of the oral cancer dataset in order to determine the accuracy of the results. This is a crucial step involved in the cancer prognosis research as the more accurate the predicted result is, the higher the feasibility of applied GP in the prognosis of cancer especially in the small dataset.

In this research, GPLAB (Silva & Almeida, 2003) is used. GPLAB is a genetic programming toolbox, which runs in the MATLAB environment. GPLAB provides GP function to different users at various levels of depth and insight as the architecture of GPLAB is highly modular and parameterized in structure. There are three main operation modules in GPLAB (GEN POP, GENERATION, and SET VARS). By manipulating the three modules, users can control the path of execution of the program and thus obtain the best results.

In addition, an amended type of GP known as Operational Equalisation Genetic Programming is also included in this research. Operator Equalisation (OpEq) solves the problems of bloat caused by the GP (Vanneschi & Silva, 2009; Silva, Dignum & Vanneschi, 2012). Bloat is the situation where the codes of GP during the evolutionary process is overgrown without any improving of the value of fitness and thus will then cause an overfitting problem. OpEq controls the size distribution in the population by probabilistically accepting every member based on their size while considering the target distribution for the probability calculation. In other words, it filters each individual in the population based on their size, length and fitness and decides whether to remain or to eliminate them based on the threshold set. However, the accuracy obtained from the GP that runs with OpEq is not stable as OpEq limits the functions of the GP and causes the features selected during the run of OpEq to be incomplete.

The GP parameters used in this study are showed in Table 2. The study was carried out by using population that ranged between 15 individuals to 100 individuals. However, the results obtained showed that the population with 31 individuals had a more stable results (i.e., less outlier individual with extreme fitness value during the final generation). In addition, a maximum generation of 5 was used in this study as the average fitness of the members in the population became constant after 5 generations.

**Table 2:** Parameters used in this research.

**GP parameters**	
Population size	31
Population initiation	‘rampedinit’
Maximum number of generation	5
Selection method	Tournament (size = 0.0100)
Crossover rate	0.01
Mutation rate	0.01
**SVM parameters**^a^	
Types of kernel	Radial basis function
gamma (*γ*) parameter	0.06
cost (*c*) penalty	1
epsilon parameter	0.001
Weight (*w*) vector	1

**Notes.**

a SVM parameter: gamma (*γ*) parameter determines the boundary of RBF kernel in which the kernel will be exceed a certain value; cost (*c*) penalty function to control the tradeoff between the two requirements, i.e., the margin of the SVM hyperplane depends on the *c* penalty; epsilon parameter determines the level of accuracy of the function; weight (*w*) parameter is an *n*-dimensional coefficient vector which is normal to the hyperplane

### Support Vector Machine

Support Vector Machine (SVM) (Vapnik, 2000) is a classification method that performs task by constructing a multidimensional space of hyperplanes that separate cases of different class labels. SVM is associated with learning algorithms that analyze data and recognize pattern, and support regression and classification tasks that can deal with multiple, continuous and categorical variables. Generally, SVM apply the kernel methods to produce a high dimensional data and construct the maximum-margin hyperplane. SVM works well with high dimensional microarray dataset. The goal of SVM is to find separating hyperplane with the largest margin (Hsu, Chang & Lin, 2003; Rosset et al., 2004; Huang & Kecman, 2005). Table 2 shows the parameters of SVM used in this research.

#### Logistic Regression

Logistic Regression (Scott, Hosmer & Lemeshow, 1991) is a predictive analysis that used to generate a linear combination of predicted outcome variables. Generally, logistic regression assumes that the log of the odds of the results is predictors’ linear function and it also estimate the function coefficient using the maximum likelihood (Magder & Hughes, 1997). The known value of binary outcome and predictors are required by this model.

## Results

Due to the automatic feature selection of GP, the features selected by GP in each run may not be the same. Therefore, in order to study the influence of each feature towards the prediction, a two-step method was used in this research.

**Figure 2:**
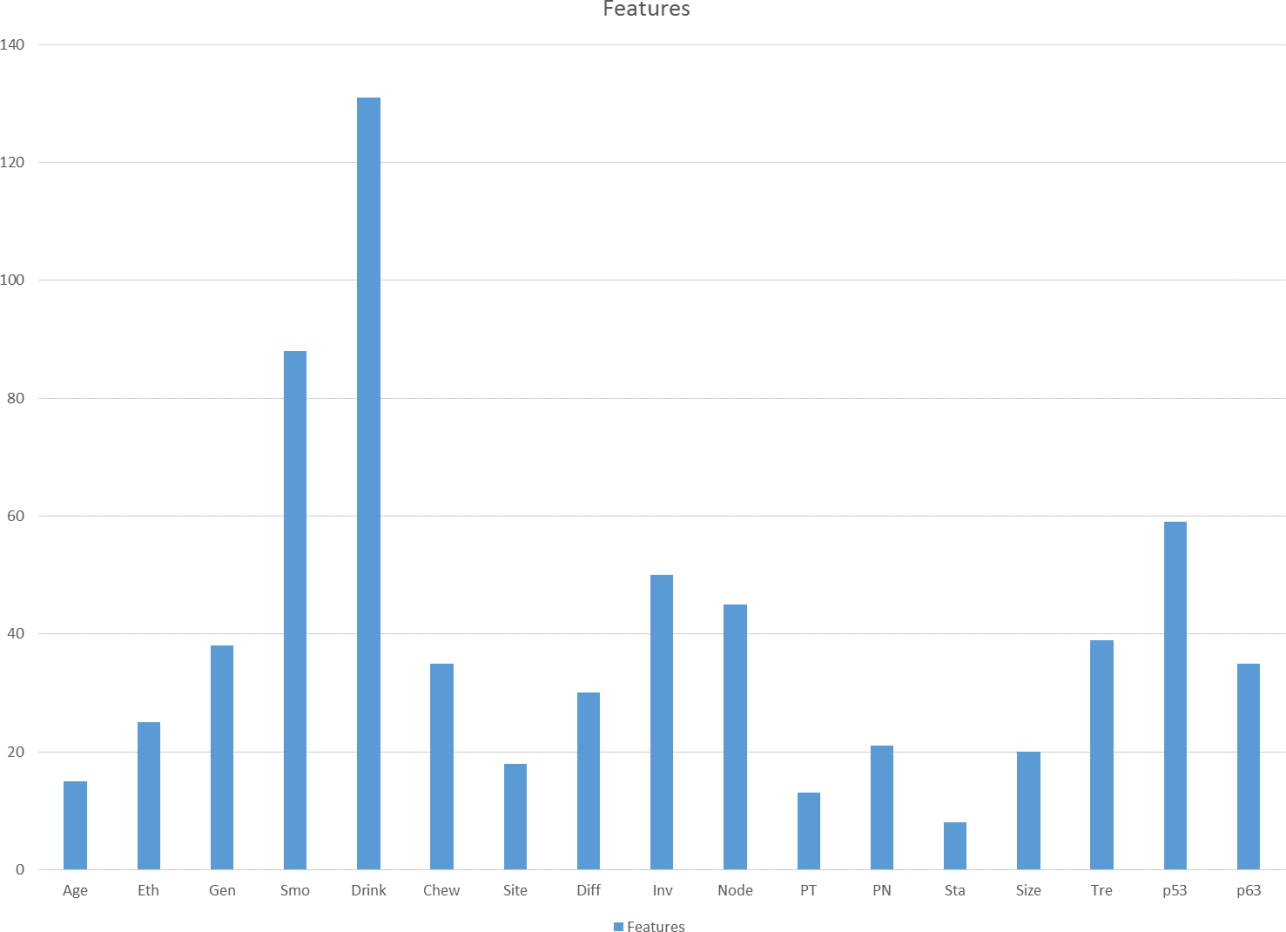
Frequency of each feature selected by GP in 20 runs.

First, the GP was tested for 20 times using all the features in the dataset and the frequency of the feature selected in 20 runs was calculated. Higher frequency of features selected by GP in 20 runs indicates that the features hold a more important rank in the prediction compared to the others. Figure 2 shows the frequency of features selected by the GP in 20 runs. The top 10 features were selected for step 2. The 10 selected features are *Dri, Smo, p53, Inv, Node, Tre, Gen, p63, Chew* and *Diff*.

In step 2, the top 10 features selected from step 1 were tested by using normal GP and OpEq GP. In this step, the automatic feature selection of GP was used in order to select the optimal subset of features. In this study, a 5-fold cross-validation is applied on the dataset. Cross-validation (Kohavi, 1995; Varma & Simon, 2006) is a well-known method used to reduce bias that may cause by small sample size. The results of the GP were averaged in order to obtain the average accuracy, and error rate. Receiver operating characteristic (ROC) curves were plotted for each feature subset and the area under the curve (AUROC) were calculated. Table 3 shows the best results of feature subsets using normal GP and OpEq GP.

**Table 3:** Best results on selected feature subsets using normal GP and Operator Equalisation GP.

No. of feature	Feature subset	Normal GP			OpEq GP		
		Average accuracy (%)	Root Mean Square Error (RMSE)	Average AUROC	Average accuracy (%)	Root Mean Square Error (RMSE)	Average AUROC
10	*Eth Smo Dri Chew Diff Inv Node Tre p53 p63*	67.74	0.4675	0.6477	67.74	0.5957	0.6559
9	*Eth Smo Dri Chew Diff Inv Tre p53 p63*	77.42	0.5165	0.7432	69.68	0.4069	0.6995
8	*Eth Smo Dri Chew Diff Inv p53 p63*	67.74	0.6947	0.6682	64.19	0.4478	0.6648
7	*Eth Smo Dri Chew Diff Inv p63*	80.65	0.5681	0.7786	78.39	0.6956	0.7589
6	*Smo Dri Chew Diff Inv p63*	64.52	0.5957	0.6432	63.87	0.5191	0.6300
**5**	***Smo Dri Chew Diff p63***	**83.87**	**0.4160**	**0.8341**	**81.29**	**0.5602**	**0.8100**
4	*Smo Chew Diff p63*	67.74	0.4600	0.6886	57.42	0.5680	0.6270
3	*Smo Diff p63*	67.74	0.5598	0.6886	60.32	0.3548	0.6414
2	*Smo p63*	64.52	0.5161	0.5000	60.65	0.4366	0.5150

**Figure 3:**
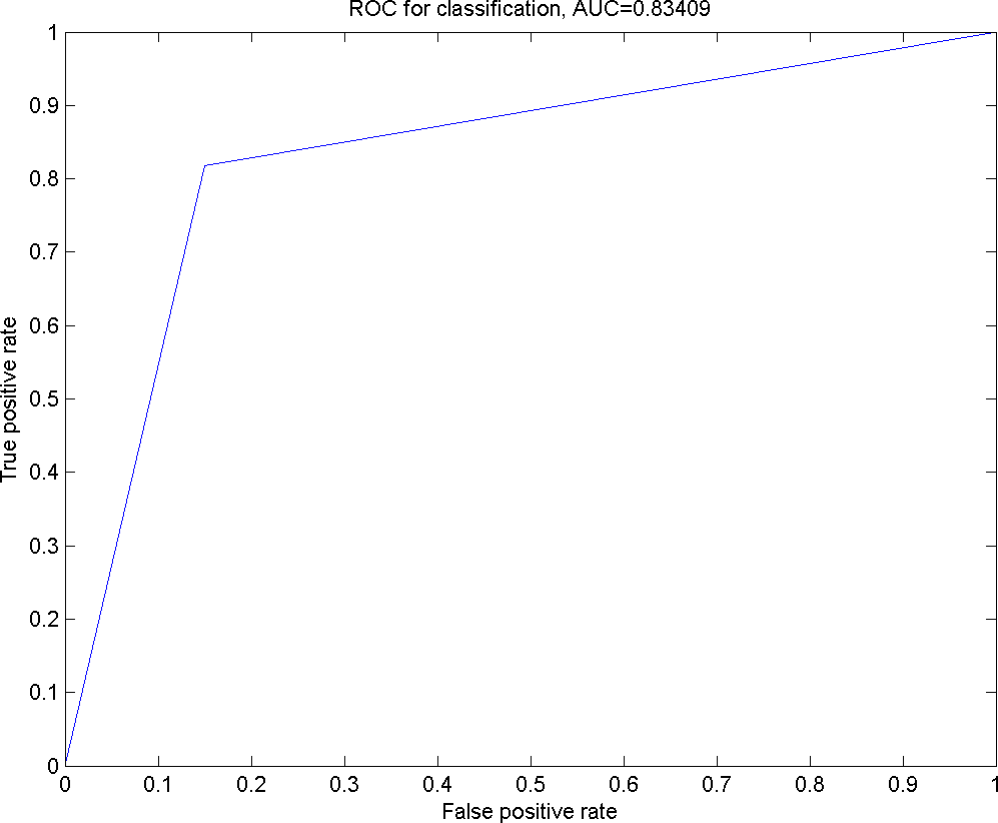
The ROC curve for the classification using Smo Dri Chew *Diff* p63 as the selected features.

In Fig. 3, an ROC curve was plotted using the true positive rate (sensitivity) against the false positive rate (fall-out). From the graph, it can be seen that the calculated AUROC is 0.83409. This suggests that the GP has successfully resulted in a higher accuracy in the prediction of the given oral dataset with *Smo, Dri, Chew, Diff* and *p*63 as the optimal feature subset.

Besides that, each feature subset in Table 3 were tested with SVM. Table 4 shows the best results of SVM on the selected feature subsets. From Table 4, it can be seen that the performance of SVM is slightly inferior compared to GP in oral cancer prediction as the best accuracy achieved is merely 64.76%, and there are not much differences in between the selected feature subsets.

**Table 4:** Best results of SVM on the selected feature subsets.

No. of feature	Feature subset	SVM		
		Average accuracy (%)	Root Mean Square Error (RMSE)	Average AUROC
10	*Eth Smo Dri Chew Diff Inv Node Tre p53 p63*	64.76	0.4000	0.5000
9	*Eth Smo Dri Chew Diff Inv Tre p53 p63*	64.76	0.4000	0.5000
8	*Eth Smo Dri Chew Diff Inv p53 p63*	64.76	0.4000	0.5000
7	*Eth Smo Dri Chew Diff Inv p63*	64.76	0.4000	0.5000
6	*Smo Dri Chew Sub Diff p63*	61.43	0.6000	0.4750
5	*Smo Dri Chew Diff p63*	64.76	0.4000	0.5000
4	*Smo Chew Diff p63*	64.76	0.4000	0.5000
3	*Smo Diff p63*	64.76	0.4000	0.5000
2	*Smo p63*	61.43	0.4000	0.5000

Besides that, the performance of GP is also compared with the performance of the simple statistical method, which is logistic regression, using the same data. Combinations of feature subsets that ranged from 2^∧^17 until 10^∧^17 were tested accordingly in this study, in order to obtain the subsets of features with the best accuracy, AUROC and also the lowest root mean square error (RMSE). Table 5 shows the best result of logistic regression for the selected features subsets.

**Table 5:** Best results of logistic regression for the selected feature subsets.

No. of feature	Feature subset	Logistic regression		
		Accuracy (%)	Root Mean Square Error (RMSE)	AUROC
10	*Smo Dri Chew Site Diff Nodes PT Sta Size Tre*	64.5161	0.7315	0.5
9	*Smo Dri Chew Site Diff PN Sta Size Tre*	64.5161	0.7304	0.5
8	*Age Smo Site Sub Nodes PT PN Sta*	64.5161	0.7303	0.5
7	*Age Smo Site Nodes PT PN Sta*	64.5161	0.7303	0.5
6	*Chew PT PN Sta Tre p63*	64.5161	0.7293	0.4545
5	*Gen Chew Inv Nodes PN*	61.2903	0.7265	0.7
4	*Age Gen Chew Size*	54.8387	0.7260	0.65
3	*Eth Gen Inv*	51.6129	0.7305	0.625
2	*Inv p53*	51.6129	0.7301	0.625

## Discussions

From the results in Table 3, it can be seen that the optimal subset that obtained the best accuracy is *Smo, Dri, Chew*, *Diff and p63.*

However, it can be seen that the accuracy of the prediction dropped to 64.52% when there is an extra feature included (*Inv*) and dropped to 67.74% when there is a feature excluded (*Dri*) from the optimal feature subset of *Smo, Dri, Chew*, *Diff and p63*. This showed that these five features are correlated to each other in order to achieve the best result. Two of the features selected from this study are correspondent with the features selected by Chang et al. (2013), which are *Dri* and *p*63. This further proved that GP is suitable to use in the oral cancer prognosis with its automatic feature selection function that could identify the optimal feature subset.

As seen in Table 4, when comparing the results of the stated methods, GP performed better than the SVM in all selected feature subsets and the best result was obtained from GP. This shows that GP outperforms SVM, even though SVM has been proven to be a good predictive technique in various previous researches (Sweilam, Tharwat & Moniem, 2010; Zheng, Yoon & Lam, 2014; Majid et al., 2014). However, in the case of predicting the survival of oral cancer, SVM is not an appropriate classification tool to be used together with a small dataset.

From Table 5, the best accuracy obtained from logistic regression method is 64.5161% with AUROC of 0.5 and RMSE of 0.7303. By comparing the results of logistic regression method with GP, it can be clearly seen that the machine learning technique GP outperformed the logistic regression method when testing with small dataset.

Smoking is always associated with the poor prognosis of oral cancer. Based on previous studies, the probability of having oral cancer among smokers is higher than in non-smokers. Tobacco leaves that are used to make cigarettes contain radioactive materials and nicotine, an additive substance. Nicotine may cause cell mutation, damaging the DNA and thus causing oral cancer (Oral Cancer Foundation, 2016). Previous studies showed that some cellular functions such as mitogenic pathway activation, angiogenesis, and cell growth of various cell types, could be altered by nicotine (Xu et al., 2007; Arredondo et al., 2006). In fact, the risk of oral cancer gets even higher when the smoker is also an alcohol drinker at the same time (Wright et al., 1993; Blot et al., 1988; Vecchia et al., 1997). The association of drinking alcohol with oral cancer has been proven by many previous studies that showed that the excess intake of alcohol will decreases the endocytic activity of the buccal mucosa cell inside the mouth (Chang et al., 2013; Loyha et al., 2012). Furthermore, the chewing of betel quid is also often related to the occurrence of oral cancer, especially for the long-term chewer. It has been showed that the chemical component of the areca nut will trigger the carcinogenicity mechanism in the oral cavity (Ogden, 2005).

In a survival analysis performed by Ko et al. (1995), they have shown that histological differentiation is one of the significant factors in determining the overall survival of OSCC. In addition, previous studies have found that the differentiation of SCC is influenced by the consumption of tobacco, drinking and betel quid chewing. According to Fang et al. (2009) and Wang et al. (2015), tobacco consumption will cause the tumor cell to dedifferentiate which makes them become more aggressive. In addition, it also proved that the increased in the alcohol consumption was associated with the increased in the risk of dedifferentiation of OSCC as well (Bundgaard & Bentzen Søgaard, 1995). Other than smoking and drinking, the habit of betel quid chewing is also shown to be closely related with the histological differentiation of SCC, especially in the poorly differentiated group of SCC (Lee et al., 2008). Again, these proved that the factors are correlated to each other.

Finally, according to Chang et al. (2013) and Choi et al. (2002), the over-expression of p63 gene is often associated with the poor prognosis of oral cancer. In Muzio et al. (2005), 10 cases of normal mucosa and ninety-four cases of oral squamous cell carcinoma were analyzed with active expression of p63 expression by immunohistochemistry technique. The study suggested that p63 expression is positively associated with the grade of neoplasm differentiation which support the use of p63 in diagnostic use of oral SCC as an additional marker. However, Oliveira, Ribeiro-Silva & Zucoloto (2007) found out that p63 positive tumor inversely had a higher Disease-Free-Survival (OS) and Overall Survival (OS) in OSCC patients. In our study, p63 is selected as one of the optimum features based on the weightage of each features that correlated to OSCC. Hence, we suggested that p63 could be employed as one of the markers in oral cancer prognosis.

## Conclusion

In this study, we presented an oral cancer prognosis method using the GP approach. In addition, in order to consolidate the results obtained in our research, comparison of the prediction performance of GP, SVM and LR were also carried out. The results showed that GP stands out to be comparably better than SVM and LR with the highest accuracy of 83.87 and AUROC of 0.8341. Also, the unique features of GP in automatically selecting the features proofs that the correlation of the features are also crucial in the classification process. The optimal features that are obtained from the GP model is *Smo, Dri, Chew, Diff and p63*.

However, further studies need to be done especially on the biological perspective of the correlation between each feature to show how are these relationship can help in improving the prognosis of oral cancer.

## Additional Information and Declarations

### Data Availability

The following information was supplied regarding data availability: The raw data was made available for peer review but cannot be published because it is owned, and restricted by, the Malaysia Oral Cancer Database and Tissue Bank System (MOCDTBS).
